# CD49b inhibits osteogenic differentiation and plays an important role in osteosarcoma progression

**DOI:** 10.18632/oncotarget.21254

**Published:** 2017-09-23

**Authors:** Tingting Ren, Sajida Piperdi, Pratistha Koirala, Amy Park, Wendong Zhang, Daria Ivenitsky, Yidan Zhang, Esperanza Villanueva-Siles, Douglas S. Hawkins, Michael Roth, Richard Gorlick

**Affiliations:** ^1^ Department of Orthopedics, Musculoskeletal Tumor Center, Peking University People's Hospital, Beijing, China; ^2^ Department of Pediatrics, Division of Hematology/Oncology, The Children’s Hospital at Montefiore, Albert Einstein College of Medicine, Bronx, NY, USA; ^3^ Department of Pathology, Montefiore Medical Center, Bronx, NY, USA; ^4^ Department of Pediatrics, Division of Hematology/Oncology, Seattle Children’s Hospital, Fred Hutchinson Cancer Research Center, University of Washington, Seattle, WA, USA; ^5^ Department of Pediatrics, Children’s Cancer Hospital, The University of Texas MD Anderson Cancer Center, Houston, TX, USA

**Keywords:** CD49b, osteosarcoma, osteogenic differentiation, mesenchymal stem cells, osteoblasts

## Abstract

Osteosarcoma is a cancer whose cell of origin lies in the differentiation pathway between the mesenchymal stem cell (MSC) and the osteoblast (OB). In this study, we sought to determine if surface markers associated with osteoblastic differentiation are involved in osteosarcoma progression. cDNA expression arrays were performed on MSCs and osteoblasts to identify differentially expressed genes. The specificity of candidate genes for osteoblast differentiation was assessed through time course experiments in differentiation media with confirmation utilizing CD49b transfected MSCs. In addition, CD49b was transfected into osteosarcoma cell lines to determine its impact on cell proliferation, motility, and invasion. Finally, the expression of CD49b was assessed in osteosarcoma patient samples and correlated with survival outcomes. cDNA expression arrays identified a list of genes differentially expressed between MSCs and osteoblasts with a subset of those genes encoding cell surface proteins. Three genes were selected for further analysis, based on qPCR validation, but only CD49b was selective for osteoblastic differentiation. Forced expression of CD49b in MSCs led to delayed osteoblastic differentiation. Down-regulation of CD49b expression in osteosarcoma cell lines resulted in inhibition of their migration and invasion capacity. CD49b expression in osteosarcoma patients was associated with presence of metastases and inferior 5 year overall survival (31.4% vs. 57.4%, p=0.03). Surface proteins involved in osteosarcoma cell differentiation, such as CD49b, have the potential to serve as prognostic biomarkers, and may lead to the identification of new therapeutic targets.

## INTRODUCTION

Osteosarcoma is the most common primary malignant bone tumor in children and adolescents [1]. Significant improvements in survival have been made in osteosarcoma with multi-agent chemotherapy in addition to surgical resection; and approximately two thirds of children diagnosed with localized osteosarcoma are cured of their disease. However, since the 1980’s, improvements in the outcomes for children diagnosed with osteosarcoma have remained stagnant. New treatments and therapeutic approaches are needed, particularly for refractory and recurrent cases. A lack of targetable driver mutations in osteosarcoma, which is exacerbated by limited knowledge regarding the cell of origin, has hindered the development of novel therapeutic agents.

Osteosarcoma is defined histologically as a malignant spindle cell tumor that produces aberrant osteoid, and could potentially be derived from a cell anywhere along the differentiation pathway between human mesenchymal stem cells (MSCs) and mature osteoblasts (OBs). MSCs are pluripotent stem cells that give rise to multiple lineages, including those comprising the skeleton such as osteoblasts, chondrocytes, and adipocytes [2]. There is controversy regarding the osteosarcoma cell of origin, with some studies implicating MSCs whereas recent data have suggested that osteosarcoma arises from a more differentiated osteoblastic cell population [3–5]. It was previously show that pre-osteoblasts transformed with hTERT, SV40 TAg, and H-Ras form spindle cell tumors in mice with the production of osteoid while tumors formed by transformed MSCs formed undifferentiated sarcomas that did not produce osteoid [6]. These and other results suggest that osteosarcoma arises from cells that have begun, but not completed, the osteogenic differentiation process. Therefore, it is possible that the cell of origin is an intermediate precursor between MSC and osteoblast.

Characterizing cell surface markers expressed during the MSC to osteoblasts differentiation process may lead to identification of intermediate-stage progenitor cells, and possibly the cell of origin. In addition, factors that maintain osteosarcoma in a differentiated state capable of producing osteoid, its diagnostic hallmark, while remaining sufficiently de-differentiated to maintain its cancer properties may be involved in its pathogenesis or progression [7]. In the present study, by screening gene expression microarrays, using cDNA of MSCs and osteoblasts, together with a series of *in vitro* experiments and clinical data analyses, we demonstrate that CD49b plays an important role in osteogenic differentiation and contributes to the malignant phenotype of osteosarcoma.

## RESULTS

### Genes differentially expressed in MSCs and osteoblasts

Gene expression microarrays were performed using both MSCs and OBs. Twenty-four genes that are translated into surface proteins demonstrated differential expression when comparing MSCs and OBs (Supplementary Table 1). Among them, six genes (CDCP1, CDH6, CDH2, CD49b, ICAM1, and ITGA9) were known to play important roles in cellular adhesion and were thus selected for further evaluation. Differential expression of these candidate genes in MSCs and osteoblasts was further validated using qPCR which demonstrated up-regulation of CDH6, CDH2 and ICAM1, and down-regulation of CDCP1 and CD49b when compared from MSC to OB (Supplementary Figure 1). These genes were selected for further analysis.

### Change in CD49b expression is specific to MSC osteogenic differentiation

**Figure 1:**
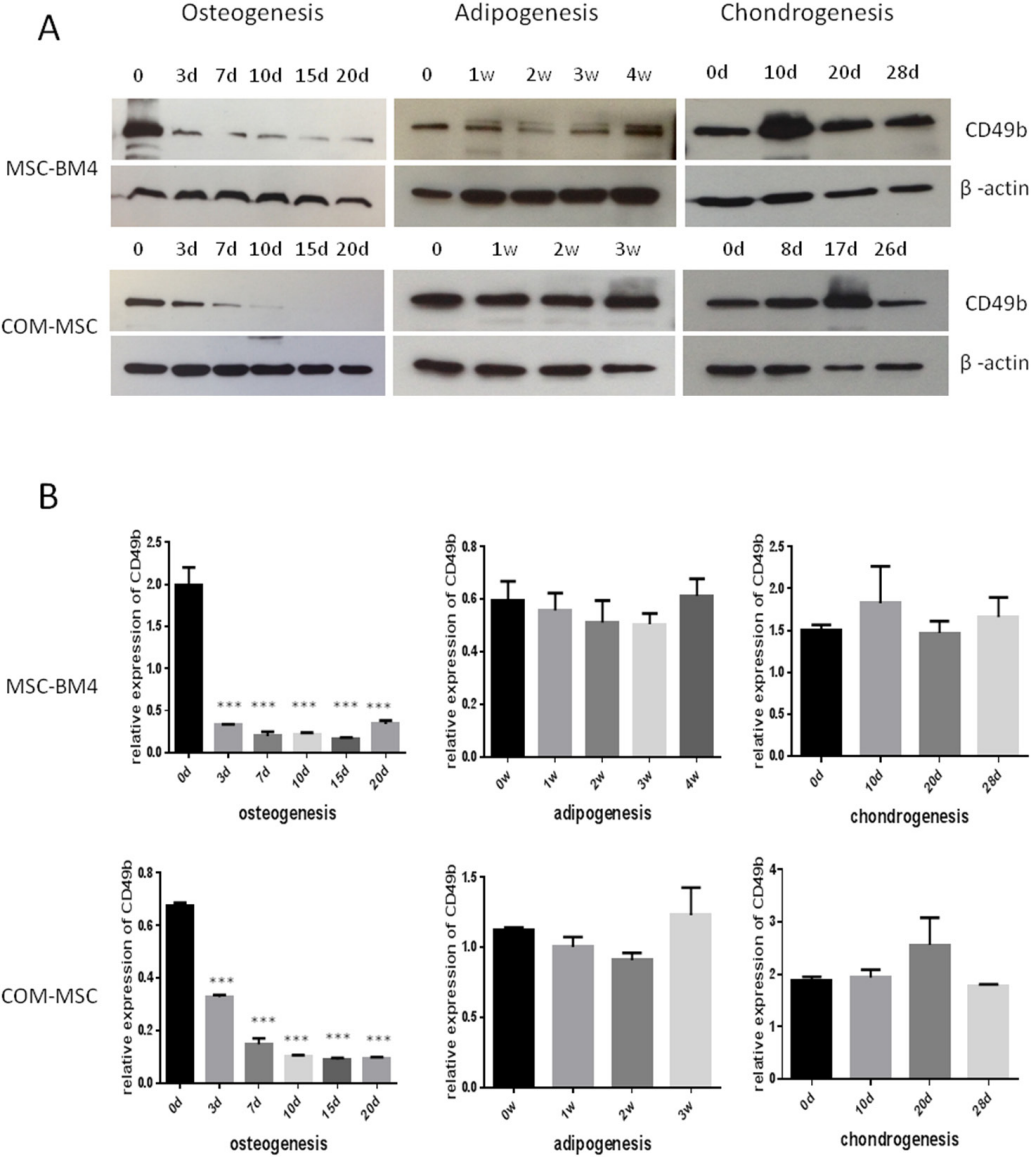
Expression of CD49b during the differentiation of MSCs into osteoblasts, adipocytes, and chondrocytes, showing only differential expression for osteogenesis, not for adipogenesis and chondrogenesis **(A)** Western blot assay was performed to detect the expression of CD49b protein, **(B)** Densitometric assays were performed and data were presented as mean ± standard deviation of three independent experiments. ^***^ means they were significantly different when compared to MSC at day 0 with p < 0.001.

The osteogenic, adipogenic and chondrogenic differentiation capacity of MSCs were assessed by Alizarin Red staining, Oil Red O staining, and immunohistochemical staining of type II collagen, respectively (Supplementary Figure 2). CDCP1 expression was down-regulated during MSCs osteogenic, adipogenic and chondrogenic differentiation (Supplementary Figure 3). CDH6 expression was up- regulated during osteogenic and chondrogenic differentiation, but remained unchanged during adipogenic differentiation (Supplementary Figure 4). No significant differences were seen in CDH2 or ICAM1 expression osteogenic differentiation (data not shown). CD49b expression, which was down-regulated during MSCs osteogenic differentiation, remained unchanged when induced into adipogenic and chondrogenic differentiation lineages (Figure 1). CD49b was the only gene that demonstrated a change in expression levels only during osteogenic differentiation, and was therefore selected for further analysis.

### CD49b inhibits MSC osteogenic differentiation via inactivation of the β–catenin pathway

**Figure 2:**
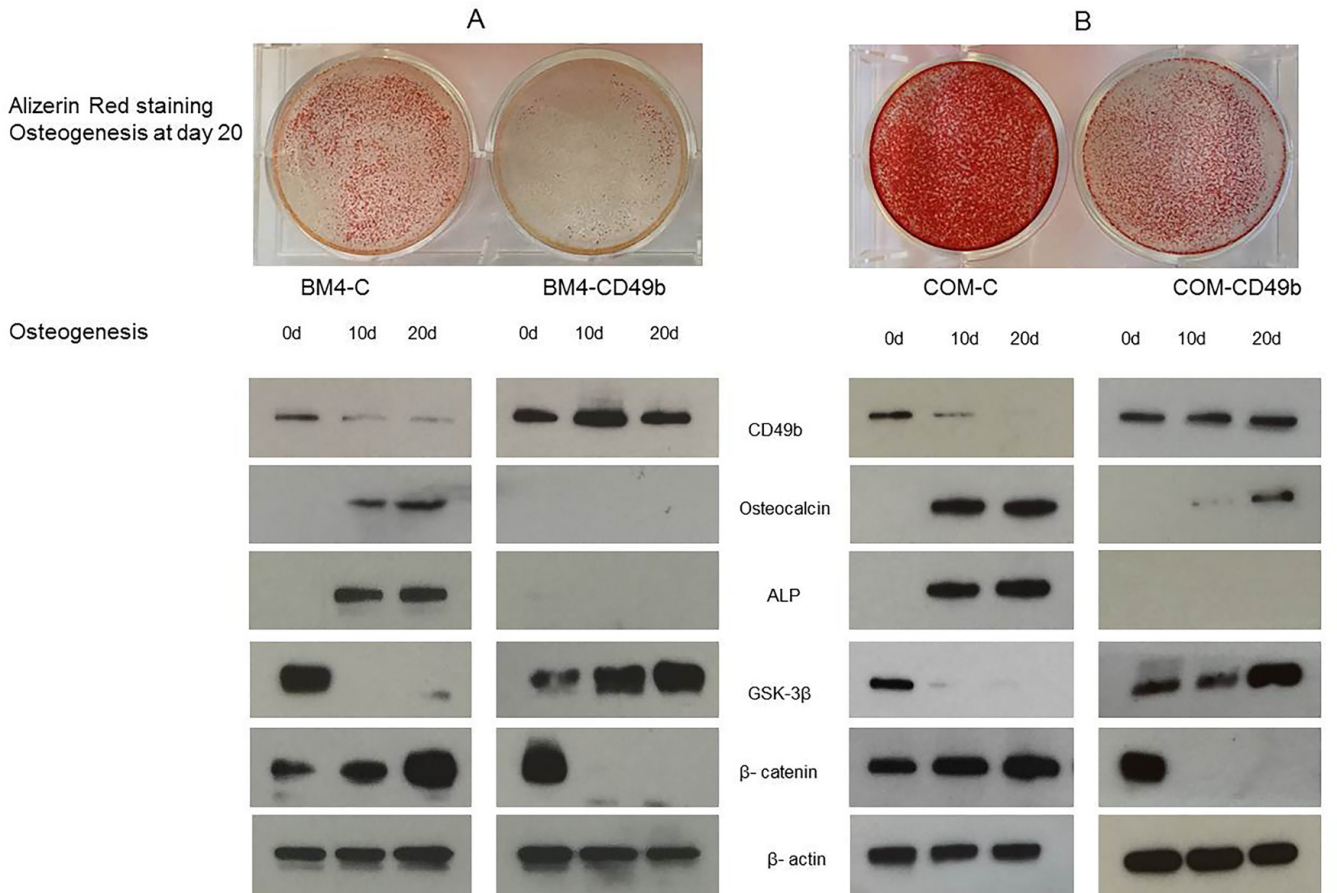
Over-expression of CD49b in MSC showing the inhibition of osteogenesis by day 20 in the transfected MSC (BM4-CD49b) compared to the control (BM4-C), decreased osteocalcin and ALP expression, increased GSK-3β, and decreased β-catenin expression **(A)** patient-derived MSC (MSC-BM4), **(B)** commercially available MSC (COM-MSC). β-actin was used as a loading control.

To further delineate the function of CD49b during MSC osteogenic differentiation, lentiviral particles were transduced to drive the expression of CD49b in MSC cells. As shown in Figure 2, expression of CD49b was up-regulated during MSC osteogenesis, whereas expression of osteocalcin and alkaline phosphatase were decreased. Alizarin red staining results showed that the production of calcium was inhibited during MSC osteogenic differentiation when CD49b was overexpressed at day 20. These findings suggest that overexpression of CD49b inhibits osteogenic differentiation. Numerous studies have previously demonstrated that the β-catenin pathway plays an important role in cell differentiation. As shown in Figure 2, expression of β–catenin was increased, while GSK-3β was decreased during MSC osteogenic differentiation. However, the transformed MSCs overexpressing CD49b demonstrated highly expressed GSK-3β and diminished β –catenin. These results suggest that the β-catenin pathway was inactivated due to the overexpression of CD49b.

### CD49b is highly expressed in osteosarcoma cells

**Figure 3:**
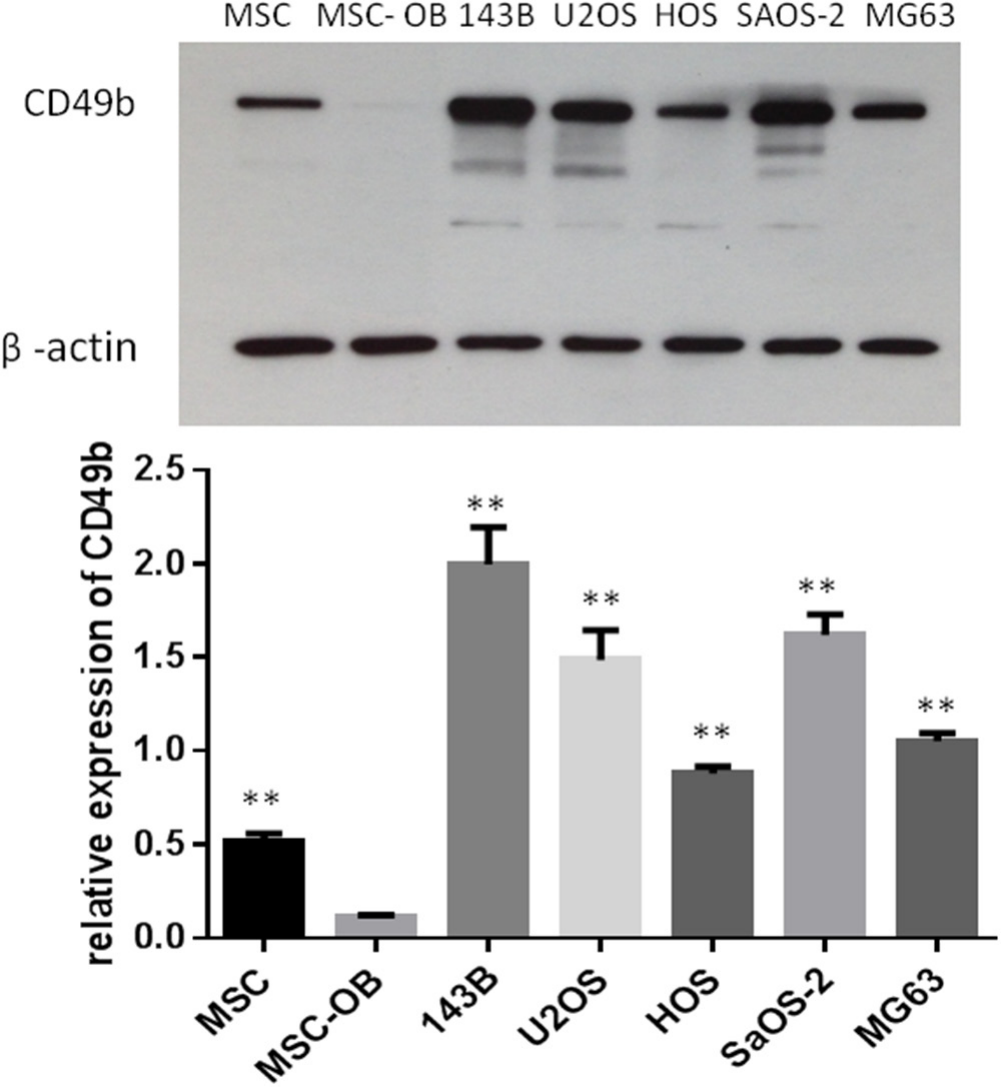
Expression of CD49b in MSC, its osteoblast derivative, and different osteosarcoma cell lines by Western blotting β-actin was used as a loading control. Data are presented as the mean ± S.D. (n=3). ^**^ means they were significantly different when compared to MSC with p < 0.01.

Expression of CD49b was assessed in MSCs, osteoblasts transduced from the MSCs (MSC-OB), and five osteosarcoma cell lines. Compared to the MSC-OB, CD49b expression was higher in all of the osteosarcoma cell lines and MSCs. Furthermore, expression of CD49b was much higher in 143B, a more aggressive cell line, than in HOS (Figure 3).

### Down-regulation of CD49b leads to inhibition of osteosarcoma cell migration and invasion *in vitro*

**Figure 4:**
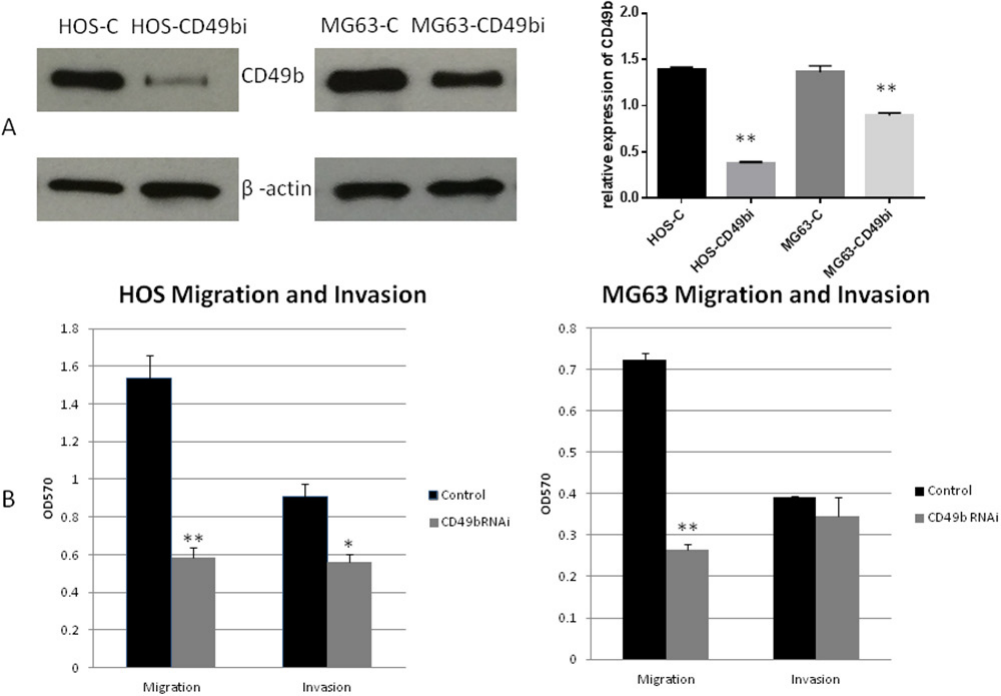
**(A)** Stable knock down of CD49b in osteosarcoma cell lines HOS and MG63. **(B)** Down regulation of CD49b in HOS and MG63 inhibits their migration and invasion capacity *in vitro*. Data are presented as the mean ± standard deviation of 3 independent experiments. ^*^ and ^**^ means they were significantly different when compared to the control group with p < 0.05 and p < 0.01, respectively.

Metastasis is one of most the critical events in tumor progression. Migration and invasion assays were performed to examine the effect of CD49b on osteosarcoma cell metastasis *in vitro*. First, expression of CD49b was knocked down in HOS and MG63 cells by transfecting a CD49b shRNA plasmid. After puromycin selection for several weeks, cell lines expressing decreased levels of CD49b were established. Western blot analysis was used to measure the expression of CD49b (Figure 4A). Migration and invasion experiments were performed using a Boyden chamber (Figure 4B). Decreased CD49b expression was associated with a significant decrease in migration in both HOS and MG63 cells. Invasion ability was reduced in HOS cells; however it was unchanged in MG63 cells. Proliferation was also examined using MTT assays. No significant difference in cell proliferation was seen in osteosarcoma cells with decreased CD49b expression (Supplementary Figure 5).

### CD49b overexpression in osteosarcoma is associated with poor prognosis in OS patients

**Figure 5:**
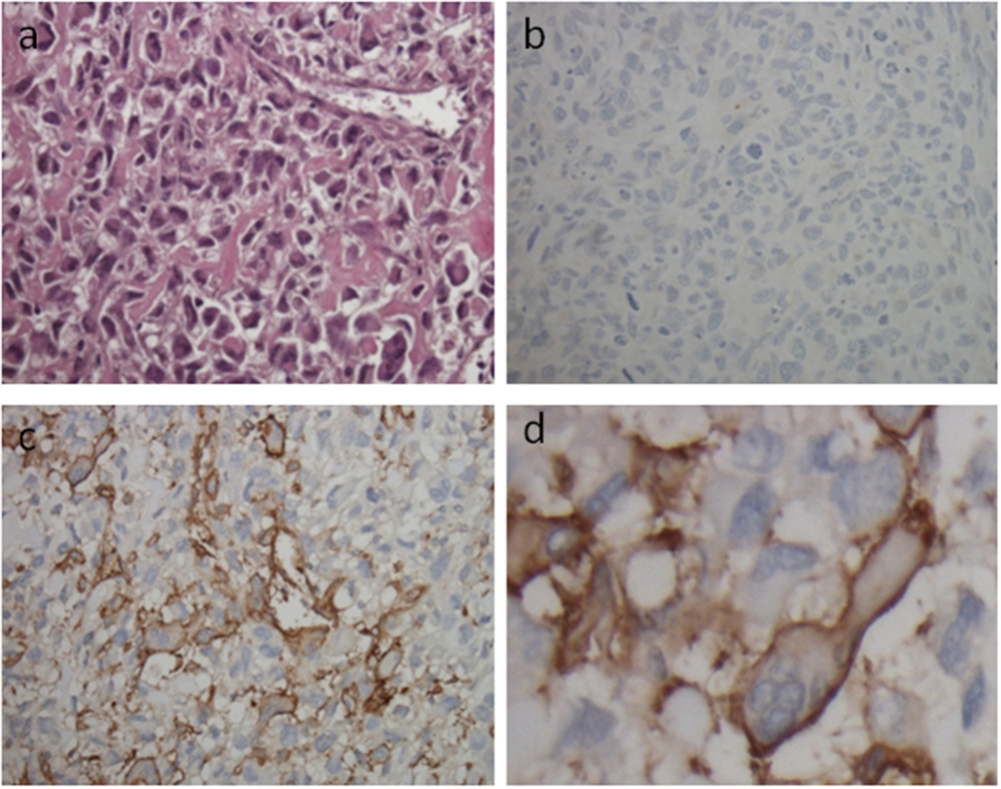
Representative pictures of immunohistochemical staining of osteosarcoma TMA punches with CD49b antibody **(a)** H&E staining is shown the osteosarcoma cells in osteosarcoma TMA (Magnification, ×100). **(b)** Negative staining of CD49b in osteosarcoma TMA (Magnification, ×100). **(c)** and **(d)** Positive staining of CD49b in osteosarcoma TMA (Magnification, ×100 and ×400, respectively). Brown staining in the membrane of osteosarcoma cells indicates CD49b expression.

CD49b expression was evaluated in a large cohort of osteosarcoma tissue samples by immunohistochemical analysis. The frequency of CD49b expression measured is described in Supplementary Table 2. A majority of samples, 61.4% (35/57), stained positive for CD49b while only 38.6% of samples were negative. CD49b protein was mainly localized in the membrane of osteosarcoma cells (Figure 5). The clinical relevance of differential CD49b expression in tumor tissues was further investigated. Clinico-pathological variables of CD49b-positive and CD49b –negative patients are summarized in Table 1. CD49b expression in tumor tissues did not correlate with age, ALP, or Huvos grade. A significantly higher proportion of osteosarcoma specimens from patients with metastatic disease demonstrated expression of CD49b compared with specimens from patients without metastatic disease (75.0% vs. 49.3%, p=0.039).

**Figure 6:**
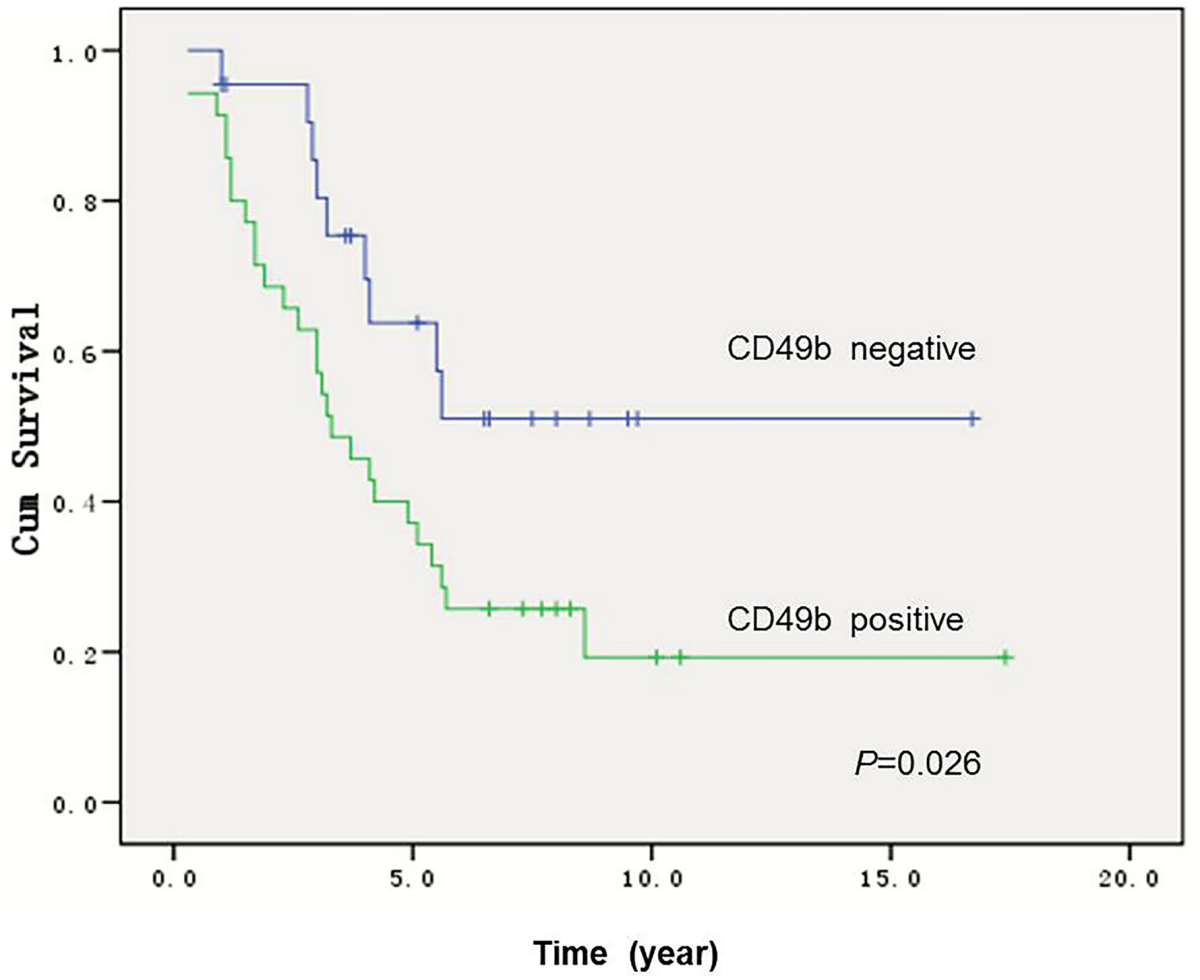
Kaplan-Meier curves for overall survival in 57 osteosarcoma patients with or without positive CD49b staining, showing that patients with CD49b–positive tumors had significantly worse overall survival (p=0.026) than patients with CD49b–negative tumors

**Table 1:** The relationship between CD49b expression and clinicopathological variables of osteosarcoma

	Clinicopathological variables	No. of cases	CD49b		*p*-value
**Negative**	**Positive**
Age	<18	37	17	20	0.125
	≥18	20	5	15	
	<90	7	1	6	
ALP	≥90	24	7	17	0.445
	unknown	26			
	<90%	20	7	13	
Huvos	≥90%	12	4	8	0.926
	unknown	25			
Lung	Yes	28	7	21	**0.039**
metastasis	No	29	15	14	

ALP: Alkaline phosphatase.

In order to determine if CD49b expression is associated with differential survival, a Kaplan–Meier survival curve was generated comparing overall survival in 57 patients based upon CD49b expression. Patients whose tumors expressed CD49b had significantly lower 5 year overall survival (31.4% vs. 57.4%, p=0.026) compared with patients with CD49b–negative tumors (Figure 6).

## DISCUSSION

Studies defining surface markers expressed on MSCs, osteoblasts, and intermediate-stage progenitor cells may help determine the cell of origin. In order to define unique surface markers expressed during the osteogenic differentiation of MSCs, gene expression analysis was performed comparing MSCs and osteoblasts. CD49b was differentially expressed and subsequently shown to be specifically involved in osteogenic differentiation. Interestingly, 4 additional genes that play a role in cell-cell adhesion, CDCP1, CDH6, CDH2, and ICAM1, were also differentially expressed during MSC differentiation, suggesting cellular adhesion and interaction with neighboring cells may play an important role in MSC differentiation. The current study demonstrated that overexpression of CD49b in MSCs inhibits osteogenic differentiation. Few studies have assessed the role of CD49b in MSC differentiation pathways. Goessler et al. previously reported that expression of CD49b remained unchanged during MSC chondrogenic differentiation which is consistent with the experimental findings in the current study [8]. The role of CD49b in MSC differentiation and progression of osteosarcoma, similarly, had not been previously investigated. Importantly, CD49b, a cell surface protein, was overexpressed in all osteosarcoma cell lines, suggesting it might be a disease marker and may be target for therapeutic intervention. To date, the majority of osteosarcoma treatments have focused on induction of cell death; targeting CD49b and its downstream pathways may represent an opportunity to instead target differentiation and metastases. Treatment approaches utilizing retinoic acid to target differentiation have a similar rationale to the proposed targeting of CD49b and have effectively improved survival in patients with neuroblastoma and acute promyelocytic leukemia [9]. Further studies are needed to validate CD49b expression in non-cancer tissue to determine its potential as a drug target.

CD49b, an integrin alpha subunit, was first identified as an extracellular matrix receptor for collagens and laminins [10]. The mechanisms by which CD49b plays a role in platelet function and homeostasis have been carefully defined via *in vitro* and *in vivo* experiments [11–12]. Prior genetic and epidemiologic studies have suggested that CD49b may play an important role in tumor progression and metastasis [13–14]. Ramirez et al. showed that in animal models of breast cancer and prostate cancer, CD49b behaved as a metastasis suppressor and decreased expression of CD49b was predictive of metastatic dissemination and poorer survival [15]. Other studies have demonstrated that increased expression of CD49b led to accelerated experimental metastasis and tumor dissemination in models of melanoma, rhabdomyosarcoma, gastric cancer and colon cancer [16–21]. While the role of CD49b in different cancers is complex, CD49b expression may be a valuable prognostic biomarker, as it is correlated with risk of metastasis and survival in various malignancies.

In order to further explore the mechanism through which CD49b expression alters the MSC osteogenesis pathway, select downstream signaling pathways were assessed in CD49b- overexpressed MSCs. Several pathways have previously been reported to be involved in MSC osteogenesis, including β -catenin signaling, Hedgehog signaling, and NELL-1 signaling [22]. β- catenin signaling and CD49b are involved in the early stages of osteogenesis and drive MSC differentiation to osteoblast progenitor cells, and for these reasons the effect of CD49b expression on the β- catenin pathway was explored [23–24]. The finding that CD49b inhibited MSC osteogenic differentiation via inactivated β –catenin pathway provides a novel approach to targeting the β –catenin pathway for therapeutic intervention. It is important to note that, while explored the impact of CD49b expression on the β –catenin pathway, additional transcription factors and signaling pathways may also be involved in CD49b regulation of osteogenic differentiation, and further studies are needed.

The current study demonstrated that CD49b involved in MSC differentiation influences osteosarcoma cell invasion and migration, two early steps in the metastasis pathway. The association between elevated CD49b and the presence of metastatic disease was also confirmed in the patient cohort. The relationship between cancer cell differentiation and the ability to metastasize has not been fully elucidated; however, prior studies suggest these two features of cancer cells may be linked. Inactivation of LKB1 in non-small cell lung cancer leads to altered cell differentiation and increased ability to metastasize [25]. Chen et. al reported that loss of glycoprotein E-cadherin in gastric adenocarcinoma is associated with poor differentiation and increased invasion into adjacent organs [26–27]. These findings suggest that targeting differentiation may decrease cancer cells’ capacity to invade locally and metastasize to distant organs.

The clinical relevance of CD49b was assessed utilizing a patient cohort that included 57 osteosarcoma samples and demonstrated that elevated expression is significantly associated with metastasis and poorer 5 year overall survival. Prior studies have demonstrated differential expression of surface proteins in osteosarcoma and ongoing studies are evaluating the prognostic implications of expression of these proteins [28]. Highly prevalent surface proteins whose expression is associated with poor survival, such as CD49b, may represent ideal targets for antibody mediated therapy. Similar strategies, such as targeting HER2 and CD20 in breast cancer and lymphoma, respectively, have led to improved outcomes [29–30]. The current study is limited by sample size and additional patient cohorts are needed to confirm the prognostic relevance of CD49b in osteosarcoma. Our studies demonstrated that CD49b may play an important role in osteosarcoma metastasis. The mechanism through which CD49b promotes local tumor growth and metastasis in osteosarcoma has not been fully elucidated, however, prior studies have demonstrated clear roles of gains and losses in specific integrins during tumor growth and dissemination in other cancers. Integrins play key roles in cancer cell migration, cell-cell adherence, and connections with endothelial cells [31], and the role of CD49b in developing cellular interactions between osteosarcoma cells and the bony matrix, as well as endothelial cells likely contribute the dissemination of osteosarcoma. Prior studies suggest that epithelial-mesenchymal transition, changes in cell-cell adhesion and the MMP2/9 ratio play important roles in the invasiveness of osteosarcoma, however, the interaction between CD49b with each of these processes needs to be specifically assessed in osteosarcoma specimens and pre-clinical models. [32–34].

In summary, this is the first study to identify surface protein CD49b as a unique marker driving MSC osteogenic differentiation and to characterize its role in osteosarcoma progression. CD49 has the potential to serve as a targetable prognostic biomarker and it may serve as a therapeutic target for therapy given its high prevalence and relevance to osteogenic differentiation. Additional prospective studies are needed to confirm the prognostic utility of CD49 expression in patients with osteosarcoma. Further studies validating consistent expression in osteosarcoma and lack of essential function in normal cells are required to define the utility of CD49b as a therapeutic target.

## MATERIALS AND METHODS

### Cells and reagents

Commercial MSC were purchased from American Type Culture Collection (ATCC, Manassas, VA) and passaged for fewer than six months after purchase. In addition, the MSC-BM4 line came from a patient undergoing a bone marrow aspirate for clinical purposes which was determined to be within normal limits by the Department of Pathology at Montefiore Medical Center. The bone marrow sample was a fully de-identified, discarded sample, therefore the laboratory analyses was determined to be exempt research by the Montefiore Medical Center Institutional Review Board. Adherent cells obtained from the bone marrow aspirate were cultured in Mesenchymal Stem Cell Medium (Lonza, Allendale, NJ). All cells were characterized either at passage 5 or 7 through Fluorescence Activated Cell Sorting (FACS) analysis. The phenotypes were uniform among all the different cells tested and in agreement with those reported for MSCs, that is, CD90, CD105, CD166, HLA-A/B/C positive (>95%), and CD34, CD 45, CD31, CD80 and HLA-DR negative (<5%). Furthermore, all hMSC cell lines were tested for their pluripotency under proper conditions towards adipocytes, chondrocytes and osteocytes. All osteosarcoma cell lines (HOS, MG-63, 143B, Saos-2, U-2OS) were obtained from ATCC (Manassas, VA) and cultured in Eagle’s Minimum Essential Medium with 10% FBS. The cell lines were maintained in a humidified chamber with 5% CO_2_ at 37°C.

Antibodies to CD49b (ab133557), CDH6 (ab133632), CDH2 (ab98952), ITGA9 (ab140599), collagen II (ab85266), osteocalcin (ab133612), alkaline phosphatase (ab97384) were purchased from Abcam (Cambridge, MA). Antibodies to CDCP1 (4115), β –actin, β –catenin (8480), GSK-3 β (12456) and horseradish peroxidase-conjugated goat anti-mouse or rabbit antibodies were purchased from Cell Signaling Technology (Danvers, MA). The dilution of the antibodies has been listed: CD49b:1/10000, CDH6:1/2000, CDH2:1/1000, ITGA9:1/2000, collagen II: 1/1000,

Osteocalcin: 1/1000, Alkaline phosphatase: 1/1000, CDCP1:1/2000, β –catenin: 1/1000, GSK-3 β: 1/1000.

### Gene expression analyses

Total RNA was extracted from MSC and osteoblasts, differentiated from the same MSC in osteoblastic differentiation media (Lonza, Allendale, NJ) for 3 weeks, using PureLink RNA Mini Kit (Life Technologies, Grand Island, NY) according to the manufacturer’s instruction. Gene expression was analyzed using Affymetrix Human genome 133A expression arrays (Affymetrix, Santa Clara, CA). Data were analyzed with Affymetrix Genechip Operating Software. All microarray assays and data analysis were performed by Genomics and Computational Genomics Core Facility at the Albert Einstein College of Medicine.

### RNA extraction and quantitative PCR (qPCR)

RNA was extracted from cell lines using Trizol reagent and was converted into cDNA using Superscript II transcriptase (Life Technologies, Grand Island, NY). qPCR was performed using a 7500 Fast Real-Time PCR system and commercially available Taqman Gene Expression assay mix (Life Technologies, Grand Island, NY; Assay IDs: CDCP1: Hs01080405_m1, CDH6: Hs00191832_m1, CDH2: Hs00983056_m1, CD49b: Hs00158127_m1, ICAM1: Hs00164932_m1, and ITGA9: Hs00979865_m1). Relative gene expression was normalized to an internal control, GAPDH, and differential expression between MSCs and OBs was calculated using the 2^−ΔΔCT^ method. Reactions for each sample were performed in triplicate. Multiple wells of scrambled control were included as negative controls. HELA, CaCo2, and PBMC cell lines were used as calibrator cell lines.

### Differentiation assays

The osteogenic, adipogenic and chondrogenic differentiation capacity of MSCs was measured by incubation in differentiation media and staining in accordance with manufacturer’s protocols (Lonza, Allendale, NJ). Cells were cultured in differentiation induction medium for 3 weeks. Differentiated cells were stained with Alizarin Red, Oil Red O and immunohistochemical staining of type II collagen using antibody collagen Type II, which stain calcium, fat, and type II collagen, respectively, to verify formation of osteocytes, adipocytes, or chondrocytes. Photos were taken using a Nikon inverted microscope ECLIPSE TE200 attached to a CCD camera (Diagnostic Instruments, Sterling Heights, MI).

### Western blot analysis

Cellular lysates were prepared by suspending 1 × 10^6^ cells in 200 μL of lysis buffer. Protein concentrations were measured using the Protein BCA Assay (Pierce, Rockford, IL). For immunoblotting analysis, equal amounts of proteins were loaded onto SDS-PAGE gels for electrophoresis and then transferred to nitrocellulose blotting membranes (GE Healthcare, Port Washington, NY). The membranes were incubated at 4°C with primary antibodies respectively. The blots were probed with the ECL Western blot detection system (GE Healthcare, Port Washington, NY).

### Plasmids and lentivirus

Lenti cDNA clone of human CD49b (RC212617L1) and Lenti-Vpak lentiviral Packaging Kit (TR30022) were purchased from Origene (Rockville, MD). The lentiviral particles were produced by transfecting the Lenti plasmid into HEK293T cells. The lentiviral particles were then tranduced into target cells and CD49b expression was examined using Western blotting. A CD49b human shRNA plasmid kit (TG312097) was purchased from Origene (Rockville, MD). CD49b shRNA plasmid or control plasmid was introduced into HOS or MG63 cells with Turbofectin 8.0 using manufacturer’s protocols for transient transfection followed by colony selection after treatment with 1 μg/ml puromycin.

### Cell migration and invasion assay

Migration and invasion kits were purchased from EMD Millipore (Billerica, MA). Cell migration assays were carried out using tissue culture-treated 6.5 mm Boyden chambers with 8.0 μM pore membranes. Cells were re-suspended in serum-free medium and transferred onto the top chamber of each transwell apparatus at a density of 1-2×10^6^ cells/ml (100 μL per chamber). Cells were allowed to migrate for 24 hours at 37°C. After removing the cells that remained in the top chamber, the top surface of each membrane was cleared of cells with a cotton swab. Then, invasive cells were stained for 20 minutes and dissolved by 10% acetic acid and measured by colorimetric reading of absorbance at 560 nm. The invasion assay was performed in the same manner as the above described migration procedure but the upper side of the membranes was coated with a uniform thickness of matrigel.

### Patient samples

Two tissue microarray (TMA) slides were utilized. One was constructed at the National Cancer Institute using samples obtained from Memorial Sloan-Kettering Cancer Center and the Children’s Hospital at Montefiore at the time of initial biopsy, definitive surgery, or at disease recurrence, as described previously [35]. A second TMA was constructed at Seattle Children’s Hospital and included tumor tissue obtained at the time of initial diagnosis (from either the primary or metastatic site), at the time of definitive surgery, and at the time of disease recurrence (either local or metastatic) with several paired samples included on the array, as previously described [36]. The samples were obtained and the TMAs were constructed and utilized in accordance with IRB approved protocols at all involved institutions. Some specimens became disadherent and were lost during the staining process; the results presented include only the samples that remained adherent and could be graded.

### Immunohistochemical analysis

Immunohistochemical staining of CD49b was performed by an experienced technician at the Albert Einstein Pathology Core Facility. Slides were baked at 60°C overnight and sections were subsequently deparaffinized and rehydrated. Endogenous peroxidase activity was blocked using 3% hydrogen peroxide for 10 minutes at room temperature. After blocking with 5% skim milk, sections were incubated with the anti-CD49b antibody at 4°C overnight followed by incubation with the secondary antibody from the En-VisionTM kit (Dako Cytomation, Carpinteria, CA) for 30 minutes at room temperature. The reaction product was visualized with diaminobenzidine (Sigma, St. Louis, MO) for 5 minutes at room temperature. Sections were counter-stained with hematoxylin. Purified IgG from normal mouse serum was used as a negative control. CD49b immunoreactivity was evaluated independently by two experienced pathologists (E. V-S, K. S (see acknowledgment)) without any knowledge of the clinical data. Tissue samples were assessed in a consecutive manner to ensure maximal internal consistency. Both the percentage of positive cells and the intensity of cytoplasmic staining were assessed in 10 randomly chosen microscopic fields. Slides were scored as negative if <10% of tumor cells were stained and positive if ≥10% of tumor cells were stained. Staining intensity was classified as follows: 0, no staining or staining in <10% of tumor cells; 1+, weak to moderate staining in 10 to 20% of tumor cells; 2+, strong staining in 10 to 20% of tumor cells or weak staining in 20 to 50% of tumor cells; 3+, moderate to strong staining in 20 to 50% of tumor cells or staining in ≥ 50% of tumor cells.

### Statistical analyses

The association between CD49b expression and clinic-pathologic variables was analyzed with the Chi-square test. Survival curves were estimated using the Kaplan-Meier method and compared with the log-rank test. All statistical analyses were performed with the SPSS statistical software package 15.0 (SPSS, Inc., Chicago, IL). P values less than 0.05 were considered statistically significant.

### Abbreviations

Mesenchymal stem cell (MSC), Tissue Microarray (TMA), Osteoblast (OB).

## SUPPLEMENTARY MATERIALS FIGURES AND TABLES


