# Supplementary material for: CD49b inhibits osteogenic differentiation and plays an important role in osteosarcoma progression
Source: Oncotarget. 2017 Sep 23;8(50):87848–59. doi: 10.18632/oncotarget.21254 (PMC5675677; doi:10.18632/oncotarget.21254)
Supplement: Supplementary file 1 [file oncotarget-08-87848-s001.pdf]

CD49b inhibits osteogenic differentiation and plays an important role in osteosarcoma progression

SUPPLEMENTARY MATERIALS

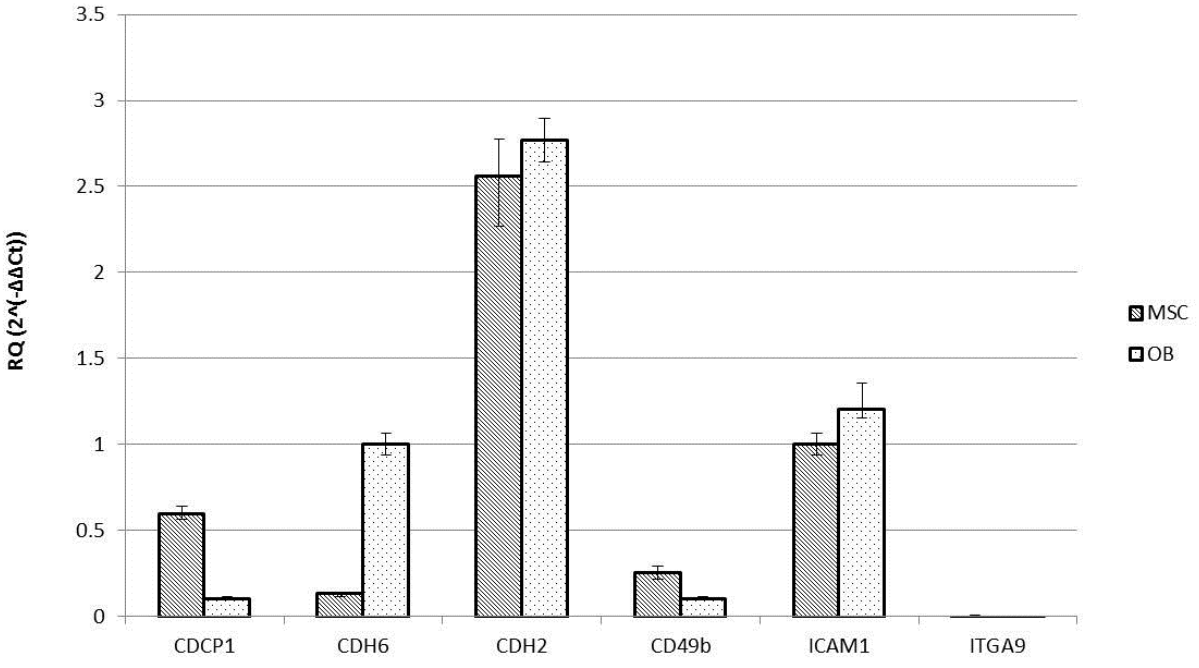

Supplementary Figure 1: Differential expression of six candidate genes were validated by quantitative real-time PCR, using GAPDH as endogenous control.

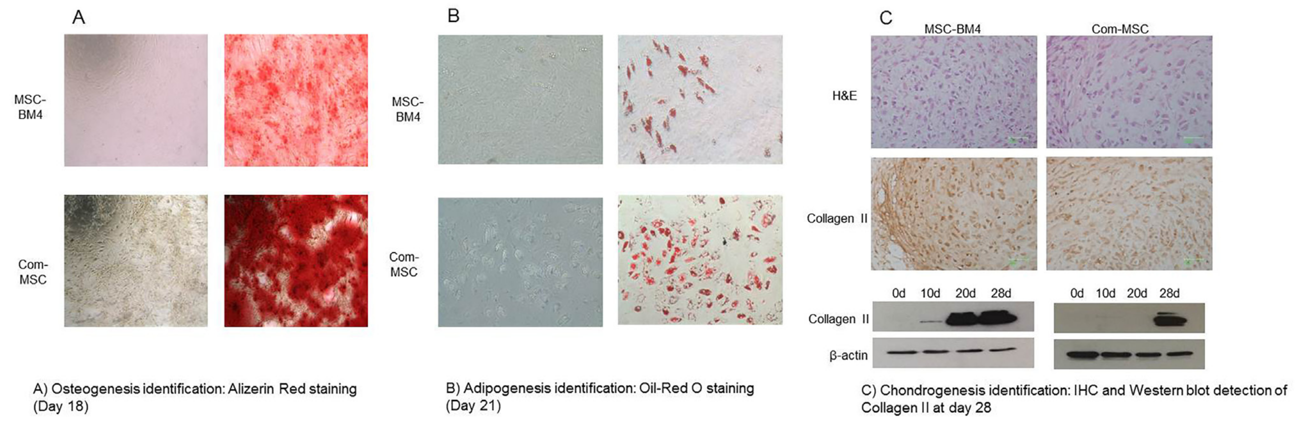

Supplementary Figure 2: Mutilineage differentiation capacity of MSCs were confirmed by **(A)** Alizarin red staining (day 18): Osteogenesis identification, **(B)** Oil-red O staining (day 21): Adipogenesis identification, and **(C)** IHC and western blot detection of collagen II at day 28: Chondrogenesis identification.

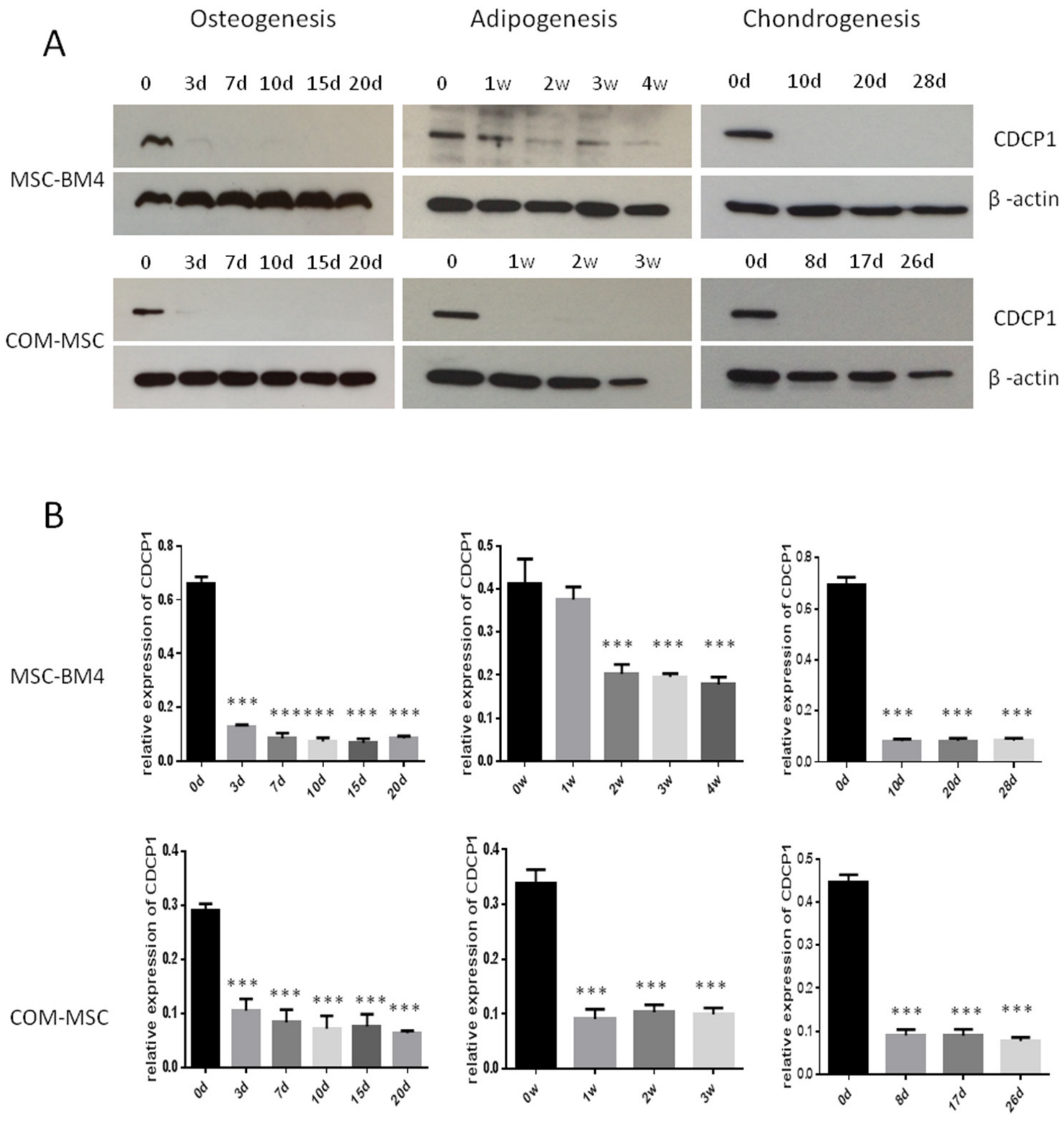

Supplementary Figure 3: Expression of CDCP1 during the differentiation of MSCs into osteoblasts, adipocytes, and chondrocytes. **(A)** western blot analysis, **(B)** densitometric analysis. ***indicates significant p-values.

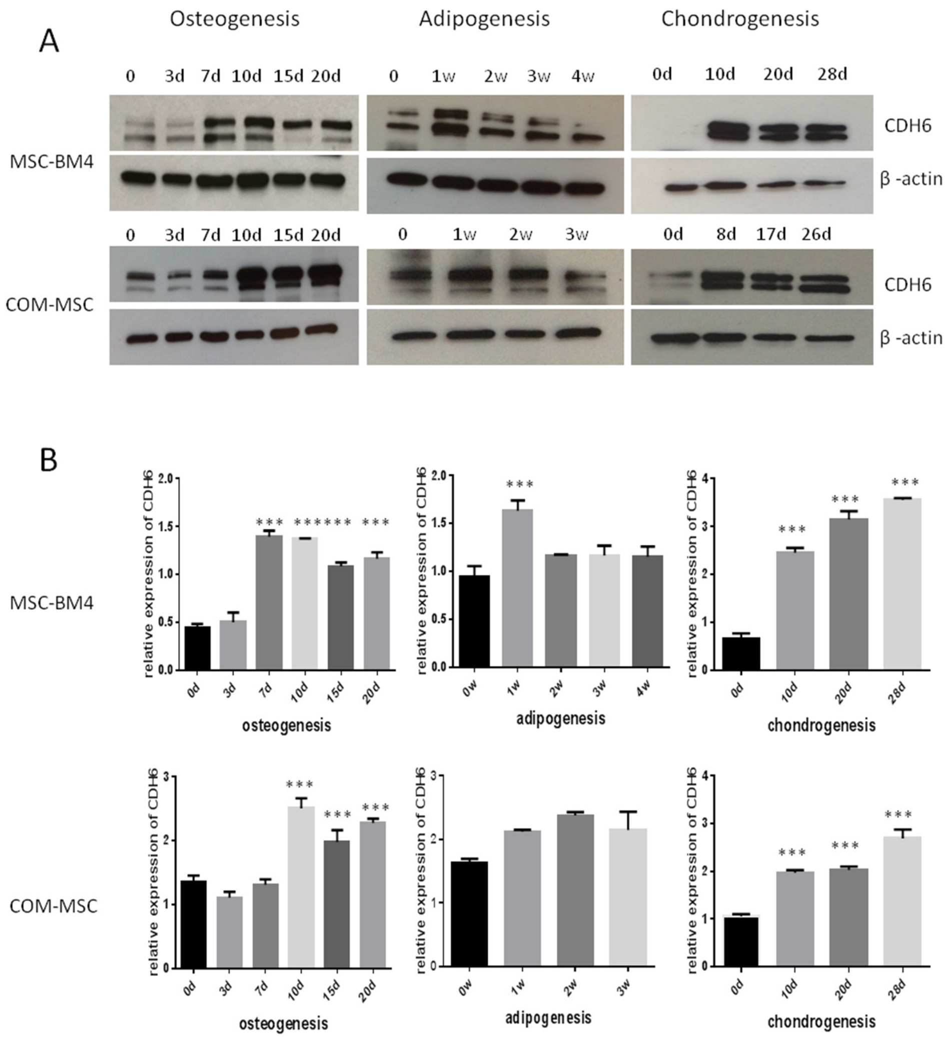

Supplementary Figure 4: Expression of CDH6 during the differentiation of MSCs into osteoblasts, adipocytes, and chondrocytes. (A) western blot analysis, **(B)** densitometric analysis. ***indicates significant p-values.

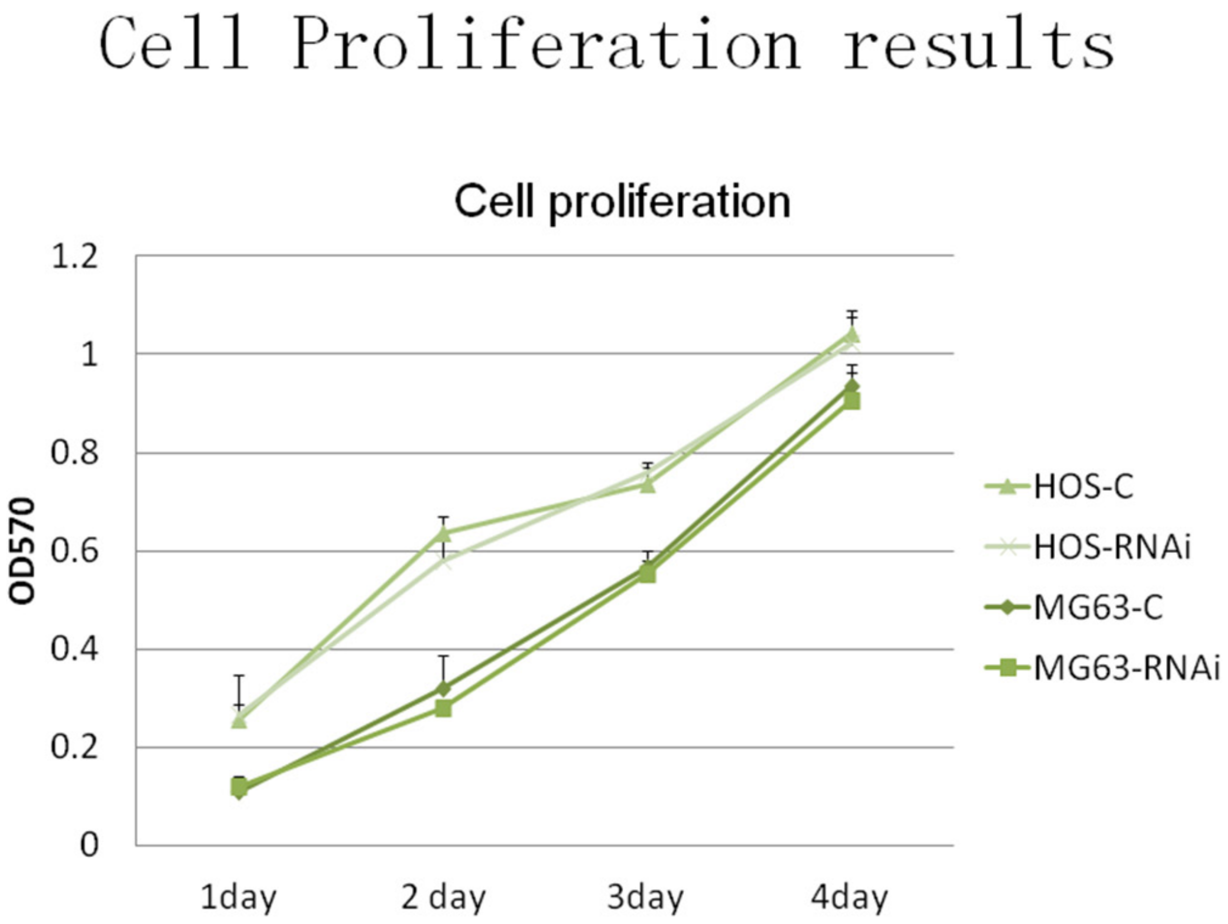

Supplementary Figure 5: Cell proliferation was examined by MTT assay. No significant difference in cell proliferation was seen in osteosarcoma cells with decreased CD49b expression.

Supplementary Table 1: Microarray - MSC and OB

genename	symbol	P.Value
Endoglin	CD105	0.005061
CUB domain containing protein 1	CDCP1	0.005073
cadherin 6, type 2, K-cadherin	CDH6	0.005315
prostate stem cell antigen	PSCA	0.006426
frizzled homolog 3	FZD3	0.00767
epidermal growth factor receptor	EGFR	0.009175
cadherin 2, type 1, N-cadherin (CD325)	CDH2/CD325	0.014005
CD72 molecule	CD72	0.019666
integrin, alpha 2 (CD49b)	ITGA2/CD49b	0.020014
CD200 molecule	CD200	0.022439
chemokine (C-X-C motif) ligand 16	CXCL16	0.022514
CD6 molecule	CD6	0.023729
colony stimulating factor 2 receptor, beta, low-affinity (CD131)	CSF2RB/CD131	0.024141
Rh blood group, D antigen	RHD	0.025365
tumor necrosis factor receptor superfamily, member 10d	TNFRSF10D	0.030534
CD1c molecule	CD1c	0.036153
CD8a molecule	CD8a	0.04123
coagulation factor II receptor-like 1	F2RL1	0.041743
intercellular adhesion molecule 1 (CD54)	ICAM1/CD54	0.041873
integrin, alpha 9	ITGA9	0.042456
CD177 molecule	CD177	0.045178
syndecan 1 (CD138)	SDC1/CD138	0.04839
dipeptidyl-peptidase 4 (CD26)	DPP4/CD26	0.048982
chemokine (C-C motif) receptor 9	CCR9	0.04952

Supplementary Table 2: Frequency of CD49b measured

Tissue	0	1+	2+	3+	Negative	Positive
	n (%)	n (%)	n (%)	n (%)	n (%)	n (%)
Osteosarcoma	22<br>(38.6)	22<br>(38.6)	11<br>(19.3)	2<br>(3.5)	22<br>(38.6)	35<br>(61.4)
